# Predictive parameters for early detection of clinically relevant abdominal trauma in multiple-injury or polytraumatised patients: a retrospective analysis

**DOI:** 10.1186/s40001-024-01969-3

**Published:** 2024-07-30

**Authors:** Stefan Fabig, Nadja Weigert, Filippo Migliorini, Jörg Kleeff, Gunther Olaf Hofmann, Philipp Schenk, Peter Hilbert-Carius, Philipp Kobbe, Thomas Mendel

**Affiliations:** 1 Department of Trauma, Hand and Reconstructive Surgery, University Hospital Jena, Am Klinikum 1, 74771 Jena, Germany; 2 Department of General, Visceral and Vascular Surgery, BG Klinikum Bergmannstrost Halle, Merseburger Strasse 165, 06112 Halle (Saale), Germany; 3 Department of Orthopaedic and Trauma Surgery, Academic Hospital of Bolzano (SABES-ASDAA), 39100 Bolzano, Italy; 4 Department of Life Sciences, Health, and Health Professions, Link Campus University, 00165 Rome, Italy; 5 Department of Visceral, Vascular and Endocrine Surgery, University Hospital Halle, Ernst-Grube-Strasse 40, 06120 Halle (Saale), Germany; 6 Department of Trauma and Reconstructive Surgery, BG Klinikum Bergmannstrost Halle, Merseburger Strasse 165, 06112 Halle (Saale), Germany; 7 Department of Science, Research and Education, BG Klinikum Bergmannstrost Halle, Merseburger Strasse 165, 06112 Halle (Saale), Germany; 8 Department of Anesthesiology, Intensive Care, Emergency Medicine and Pain Therapy, BG Klinikum Bergmannstrost Halle, Merseburger Strasse 165, 06112 Halle (Saale), Germany; 9 Department of Trauma, Hand and Reconstructive Surgery, University Hospital Halle, Ernst-Grube-Strasse 40, 06120 Halle (Saale), Germany

**Keywords:** Polytrauma, Bowel and mesenteric injury, Parenchymatous injury, Computed tomography, Laboratory parameters, Predictive parameters

## Abstract

Diagnosis of relevant organ injury after blunt abdominal injury (AI) in multiple-injury/polytraumatised patients is challenging. AI can be distinguished between injuries of parenchymatous organs (POI) of the upper abdomen (liver, spleen) and bowel and mesenteric injuries (BMI). Still, such injuries may be associated with delays in diagnosis and treatment. The present study aimed to verify laboratory parameters, imaging diagnostics, physical examination and related injuries to predict intraabdominal injuries. This retrospective, single-centre study includes data from multiple-injury/polytraumatised patients between 2005 and 2017. Two main groups were defined with relevant abdominal injury (AI^+^) and without abdominal injury (AI^−^). The AI^+^ group was divided into three subgroups: BMI^+^, BMI^+^/POI^+^, and POI^+^. Groups were compared in a univariate analysis for significant differences. Logistic regression analysis was used to determine predictors for AI^+^, BMI^+^ and POI^+^. 26.3% (271 of 1032) of the included patients had an abdominal injury. Subgroups were composed of 4.7% (49 of 1032) BMI^+^, 4.7% (48 of 1032) BMI^+^/POI^+^ and 16.8% (174 of 1032) POI^+^. Pathological abdominal signs had a sensitivity of 48.7% and a specificity of 92.4% for AI^+^. Transaminases were significantly higher in cases of AI^+^. Pathological computed tomography (CT) (free fluid, parenchymal damage, Bowel Injury Prediction Score (BIPS), CT Grade > 4) was summarised and had a sensitivity of 94.8%, a specificity of 98%, positive predictive value (PPV) of 94.5% and, negative predictive value (NPV) of 98.2% for AI^+^. The detected predictors for AI^+^ were pathological abdominal findings (odds ratio (OR) 3.93), pathological multi-slice computed tomography (MSCT) (OR 668.9), alanine (ALAT) ≥ 1.23 µmol/ls (OR 2.35) and associated long bone fractures (OR 3.82). Pathological abdominal signs, pathological MSCT and lactate (LAC) levels ≥ 1.94 mmol/l could be calculated as significant risk factors for BMI^+^. For POI^+^ pathological abdominal MSCT, ASAT ≥ 1.73 µmol/ls and concomitant thoracic injuries had significant relevance. The study presents reliable risk factors for abdominal injury and its sub-entities. The predictors can be explained by the anatomy of the trunk and existing studies. Elevated transaminases predicted abdominal injury (AI^+^) and, specifically, the POI^+^. The pathological MSCT was the most reliable predictive parameter. However, it was essential to include further relevant parameters.

## Introduction

The outcome of multiple-injury/polytraumatised patients is primarily determined by the amount and extent of the injured organ systems [1, 2]. Musculoskeletal lesions of the trunk and extremities, traumatic brain injuries, and cardiovascular or pulmonary lesions of the chest are commonly detected well by multi-slice computed tomography (MSCT) within emergency diagnostics [3–5]. However, diagnosing a relevant abdominal injury (AI) could be sophisticated [6, 7].

Generally, AI can be separated into blunt injuries of parenchymatous organs (POI) of the upper abdomen (liver and spleen) and bowel and mesenteric injuries (BMI) [8]. They are often indicated by secondary hints such as fluid or bowel gas discharge into the peritoneal cavity. Hence, they may be associated with delays in diagnosis and treatment [9, 10]. Thus, the assumption exists that the shorter the prehospital timeframe, the less distinct the indicators in MSCT, resulting in a higher risk of missing the presence of intraabdominal lesions [11].

Frequently, the mechanism of individual accidents is unclear. However, POIs are commonly caused by a sudden acceleration or deceleration of the blood-filled liver or spleen [12]. Parenchymatous disruption may lead to relevant intraabdominal bleeding followed by hypovolemic shock and coagulopathy [13]. In BMI, the high-energy impact increases intraluminal pressure, resulting in immediate perforation or a primary interruption of mesenteric blood supply, which is later followed by an ischaemia-induced secondary perforation [14, 15]. The resulting peritonitis induces a life-threatening septic shock syndrome [16]. A delay of BMI diagnosis of 5–8 h significantly increases morbidity and mortality [17, 18].

Intraabdominal injuries in multiple-injury/polytraumatised patients are seen in 15–25% of cases [12]. The following diagnostic tools are available in modern emergency room setups. The sensitivity of focused assessment with sonography for trauma (FAST) to detect free intraperitoneal fluid ranges between 77 and 88%, with a specificity of 98–99% [19, 20]. However, the detection of free abdominal fluid is not enough to detect intraabdominal damage, especially for injuries to the bowel or mesentery [21]. MSCT, as the diagnostic gold standard, shows a high sensitivity of 75–96% and specificity of 79–99% to identify intraabdominal lesions. However, false-negative results are not uncommon [22, 23]. These patients belong to a high-risk group for missing intraabdominal injuries [24]. Findings in the physical examination, such as seatbelt marks, abdominal-wall ecchymosis or haematoma, increasing abdominal pain or tenderness, distension and vomiting, can give valuable hints [21, 25]. Although these observations can increase diagnostic safety, their positive predictive value of 30–46% is relatively low for detecting hollow-organ injuries [17, 26]. Furthermore, an increased lactate (LAC) level in laboratory blood investigation is a predictor of blunt bowel and mesenteric injury. It is related to higher mortality for such patients [21, 27].

Several scoring systems have been established in the past few years to focus on early detection of BMI [26, 28]. The Bowel Injury Prediction Score (BIPS) by McNutt et al. [26] contains a system to score computed tomography (CT) findings, white blood cell count (WBC) and clinical signs of tenderness. A preliminary scoring tool (PST) developed by Raharimanantsoa et al. [28] considers parameters such as trauma mechanism, abdominal pain or tenderness, attendant long-bone fractures, LAC level and appearance of free fluid in MSCT.

Despite diverse diagnostic tools, relevant intraabdominal injuries are missed in up to 13% of cases [10]. This study aimed to verify other specific blood values of primary laboratory testing routinely taken in the emergency room (ER) for their possible validity in predicting AI. Values were compared to imaging diagnostics (MSCT, FAST) and physical examination results.

## Methods

### Study design

In this retrospective study, data were analysed from multiple-injured/polytraumatised patients who were treated in our level-I trauma centre between 2005 and 2017, following a standard ER diagnostic protocol. The study was approved by the independent ethical committee of the medical council of Saxony-Anhalt, Germany, and confirmed under approval no. 74/18, which is in line with the European data protection regulation.

### Data collection

**Fig. 1:**
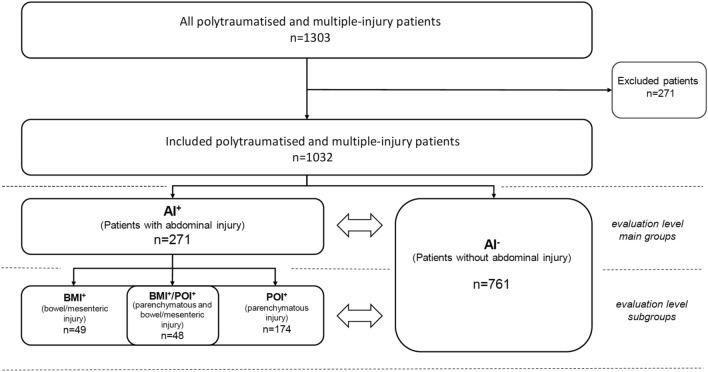
Patient recruitment. *AI* abdominal injury, *BMI* bowel and mesenteric injury, *POI* parenchymatous organ injury

Patients were identified by scanning the electronic hospital information system using an automatic search routine. Documented findings on age, trauma mechanism, American Society of Anaesthesiologists (ASA) classification, and physical examination were captured. The presence of seat belt signs, haematoma or abdominal pain or tenderness was summarised in one (dichotomous) dummy variable. The Injury Severity Score (ISS), abdominal Abbreviated Injury Scale (AIS), mortality, and duration of hospital stay were recorded. Laboratory parameters were gathered; in detail, they were: WBC, serum lactate (LAC), glucose (GLU), myoglobin (MYO), C-reactive-protein (CRP), procalcitonin (PCT), alanine (ALAT) and aspartate (ASAT) transaminases, γ-glutamyl transferase (γ-GT), alkaline phosphatase (AP). MSCT body scans were analysed for relevant findings such as bowel wall thickening, mesenteric haematoma, free intraabdominal fluid, pneumoperitoneum, active bleeding and attendant injuries of the liver and spleen. Overall evidence of pathological CT signs was summarised in a (dichotomous) dummy variable. Results of emergency FAST were categorised as pathologic with evidence of free intraabdominal fluid, POI, or both. Cases with penetrating abdominal injury without CT imaging of the trunk and laboratory diagnostics were primarily excluded (Fig. 1).

### Formation of groups

For data analysis, the cohort was divided into two main groups based on the finally documented diagnosis of either a relevant blunt abdominal injury requiring surgical intervention or clinical control and monitoring (AI^+^) or traumatised patients without abdominal injury (AI^−^). Regarding organ involvement, the AI^+^ group was divided into three subgroups: BMI^+^, POI^+^/BMI^+^ and POI^+^. Data were analysed in a main and subgroup analysis and compared to the AI^−^ reference group (Fig. 1).

### Statistical analysis

Statistical analysis was performed using the software IBM SPSS version 25 (International Business Machines Corporation, Armonk, USA). A confidence interval of 95% was assumed (significance level *p* < 0.05). Continuous data were reported as medians and interquartile range (IQR). Categorical data were indicated as frequencies and percentages. Uneven group distributions and interval scale-based variables were normalised using Z-transformation and natural log transformation. Comparing parameters between main groups was conducted using the *t*-test for transformed data. Ordinal scaled data and nominal data were compared using the Mann–Whitney-*U*-test and Pearson’s *χ*^2^-test, respectively. For dichotomous data, sensitivity, specificity, positive predictive value (PPV), negative predictive value (NPV) and odds ratio (OR) were calculated based on Pearson’s *χ*^2^-test. Univariate analysis of variance (ANOVA) was used to compare variables between the subgroups. A cut-off value of ALAT, ASAT, and LAC was estimated using receiver operating characteristic (ROC) analysis using the Youden Index for BMI^+^, POI^+^, and AI^+^ groups. Finally, the stepwise backward method of binary logistic regression modelling was used to analyse the individual power of the parameters above to predict specific POI or BMI organic injury and the AI^+^ main group. Nevertheless, data sets containing missing data were analysed, stating the number of cases.

## Results

### Descriptive data

In a 13-year time period, 1303 multiple-injury and polytraumatised patients were treated in our hospital. Two hundred seventy-one patients were excluded, resulting in a final population of 1032 patients. In the majority of the cohort (73.7%, 761 of 1032), the abdomen remained unharmed (AI^−^), but 26.3% (271 of 1032) patients had a significant abdominal injury that met the defined criteria of the AI^+^ group. Distributions according to the main and subgroups are shown in Fig. 1. The median age was 44 (27–60) years. AI^+^ patients with an age of 36 (23–52) years were significantly younger than AI- patients at 47 (29–62) years (*p* < 0.001). The most frequent trauma mechanism was car accidents (36.7%, 378.74 of 1032), followed by falls from great heights (23.3%, 240.46 of 1032) and motorcycle accidents (17.6%, 181.63 of 1032). The median Injury Severity Score (ISS) was 27 (18–35). Median ISS was significantly higher in the AI^+^ group than in the AI^−^ group. Accordingly, a significantly longer intensive care unit (ICU) stay, length of hospital stay, and a higher mortality rate were recorded. Statistical data are shown in Table 1.

**Table 1:** Evaluation of the main groups

	Sample size AI^+^|AI^−^	AI^+^	Odds ratio	AI^−^	*p*
Clinical signs					
Pathological abd. signs	271|761	48.70%	11.5	7.60%	< 0.001
Laboratory findings					
WBC (Gpt/l)	271|761	13.3 (9.6–17.6)		13.0 (10.0–17.1)	n.s
GLU (mmol/l)	200|588	8.4 (7.1–10.6)		7.4 (6.2–9.2)	< 0.001
LAC (mmol/l)	229|620	3.0 (1.9–4.7)		1.9 (1.3–2.9)	< 0.001
ASAT (µmol/ls)	267|756	2.3 (1.1–4.5)		0.8 (0.5–1.3)	< 0.001
ALAT (µmol/ls)	269|756	1.8 (0.8–3.8)		0.6 (0.4–0.9)	< 0.001
γ-GT (µmol/ls)	259|731	0.4 (0.2–0.7)		0.4 (0.2–0.7)	n.s
AP (µmol/ls)	182|510	0.9 (0.7–1.2)		1.0 (0.8–1.2)	0.002
CRP (mg/l)	264|756	3.0 (3.0–3.0)		3.0 (3.0–4.0)	n.s
PCT (ng/dl)	132|375	0.1 (0.1–0.1)		0.1 (0.1–0.1)	n.s
MYO (µg/l)	204|521	1452 (683–2653)		649 (268–1238)	< 0.001
Imaging diagnostics					
FAST: evidence of pathological signs (y/n)	214|570	68.2%	99.8	2.10%	< 0.001
CT: overall evidence of pathological signs (y/n)	271|761	94.8%	913.0	2.0%	< 0.001
BIPS CT grade ≥ 4^a^	271|761	49.8%	150.1	0.7%	< 0.001
CT: free fluid	271|761	77.9%	202.3	1.7%	< 0.001
CT: parenchymal damage	271|761	74.2%	725.5	0.4%	< 0.001
Associated injuries					
Long-bone fracture	271|761	51.3%	1.5	41.7%	0.006
Thorax	271|761	84.1%	2.7	66.5%	< 0.001
Spine	271|761	50.9%		52.6%	n.s
Pelvis	271|761	38.4%	2.0	23.4%	< 0.001
Scores					
AIS abdomen	270|761	3 (2–4)		0 (0–2)	< 0.001
ISS	270|761	3 (2–4)		24 (17–34)	< 0.001
Clinical course					
ICU (d)	270|761	12 (3–25)		6 (2–18)	< 0.001
Length hospital stay (d)	270|761	32 (16–61)		26 (14–58)	n.s
Death	270|761	15.9%		8.0%	< 0.001

Presented data follow the time sequence of in-hospital trauma management

Categorical data are reported as percentages of the corresponding main group; Continuous data are presented as median (1.Q–3.Q)

*AI* abdominal injury, *WBC* white blood cell count, *GLU* glucose, *LAC* lactate, *ASAT* aspartate transaminase, *ALAT* alanine transaminase, *γ-GT* γ-glutamyl transferase, *AP* alkaline phosphatase, *CRP* C-reactive-protein, *PCT* procalcitonin, *MYO* myoglobin, *FAST* focused assessment with sonography for trauma, *BIPS* Bowel Injury Prediction Score, *CT* computed tomography, *AIS* abdominal Abbreviated Injury Scale, *ISS* Injury Severity Score, *ICU* intensive care unit, *n.s.* no significance

^a^BIPS CT grade ≥ 4 subsumes CT findings of mesenteric contusion or haematoma, bowel wall thickening, adjacent interloop fluid collection, active bleeding, and pneumoperitoneum

Urgent abdominal surgery was performed in 60.9% (165 of 271) of AI^+^ patients. Sixteen laparoscopies and 149 laparotomies were performed. Conversion from endoscopic exploration to open procedure was necessary in 11 patients. However, continuous clinical monitoring was necessary in 39.1% (106 of 271) of AI^+^ cases.

### Main group analysis of predictive parameters

48.7% (132 of 271) of the AI^+^ group and 7.6% (58 of 761) of the AI^−^ group clinical examination revealed evidence of abdominal organ injury. Clinical examination, therefore, yielded a sensitivity of 48.7%, a specificity of 92.4%, a PPV of 70% and an NPV of 84% (OR 11.5).

Blood samples showed significantly higher values in the liver-specific ASAT and ALAT parameters in the AI^+^ group compared to the AI group. Furthermore, significant increases in non-abdomen-specific values such as GLU, MYO, and LAC were found. No differences could be detected in inflammatory parameters such as WBC, CRP, and PCT.

In imaging diagnostics, FAST could detect evidence of pathological findings in only 68% (146 of 271), conforming to a sensitivity of 68.2%, a specificity of 97.9%, a PPV of 92.4%, and an NPV of 89.1% (OR 99.8) in AI^+^ patients.

Overall, MSCT revealed signs of intraabdominal injuries in 94.8% (257 of 271) AI^+^ patients. In only 2.0% (15 of 761) AI^−^ cases, CT showed suspicious findings (sensitivity: 94.8%, specificity: 98%, PPV: 94.5%, NPV: 98.2%; OR 913). CT findings like mesenteric contusion or haematoma, bowel wall thickening, interloop fluid collection, bleeding and pneumoperitoneum were considered together, according to CT criteria of the Bowel Injury Prediction Score (BIPS) [26]. In 49.8% (135 of 271) AI^+^ patients, the BIPS value was ≥ 4. Crosstab analysis showed a low sensitivity of 49.8% but a high specificity of 99.3%. PPV and NPV were 96.4% and 84.5%, respectively (OR 150.1). Evidence of free fluid was positive in 77.9% (211 of 271) AI^+^ patients (sensitivity: 77.8%, specificity 98.3%, PPV: 94.1%, NPV: 92.5%; OR 202.3). Parenchymal damage was verifiable in 74.2% (201 of 271) AI^+^ patients (sensitivity: 74.2%, specificity: 99.6%, PPV: 98.5%, NPV: 91.5%; OR 725.5).

The incidence of associated long-bone fractures and thoracic and pelvic lesions was significantly more frequent in the AI^+^ group than in the AI group. However, the evidence of spine injuries did not show differences (Table 1).

### Subgroup analysis of predictive parameters

The AI^+^ group was divided into three subgroups: BMI^+^, POI^+^/BMI^+^ and POI^+^. At this, priority is given to comparison with the group of persons without abdominal injury (AI^−^). Statistical data are shown in Table 2.

**Table 2:** Subgroup analysis of predictive parameters

	Sample size^b^	BMI^+^	BMI^+^/POI^+^	POI^+^	AI^−^
Clinical signs					
Pathological abd. signs	49|48|174|761	73.5%	68.8%	36.8%	7.5%
Laboratory findings					
LAC (mmol/l)	37|40|152|620	2.95 (2.03–6.37)	4.27 (2.09–5.90)	2.9 (1.78–4.40)	1.90 (1.24–2.92)
ASAT (µmol/ls)	48|45|174|756	1.50 (0.69–2.55)	2.64 (1.16–6.33)	2.58 (1.38–5.15)	0.79 (0.53–1.26)
ALAT (µmol/ls)	49|47|173|756	1.23 (0.57–1.63)	1.92 (0.84–4.76)	2.1 (1.05–4.15)	0.62 (0.42–0.92)
Imaging diagnostics					
FAST: evidence of pathological signs (y/n)	34|36|145|569	67.6%	80.6%	65.5%	1.4%
CT: overall evidence of pathological signs (y/n)	49|48|174|761	93.9%	95.8%	96.0%	1.7%
BIPS CT grade ≥ 4^a^	49|48|174|761	85.7%	89.6%	28.2%	0.8%
CT: free fluid	49|48|174|761	89.8%	95.8%	70.1%	1.6%
CT: parenchymal damage	49|48|174|761	8.2%	75.0%	93.6%	0.1%
Associated injuries					
Long-bone fracture	49|48|174|761	57.1%	50.0%	50.6%	41.8%
Thorax	49|48|174|761	65.3%	87.5%	87.9%	66.6%
Spine	49|48|174|761	53.1%	56.3%	48.3%	52.7%
Pelvis	49|48|174|761	42.9%	41.7%	36.2%	23.4%

*BMI* bowel and mesenteric injury, *POI* parenchymatous organ injury, *AI* abdominal injury, *LAC* lactate, *ASAT* aspartate transaminase, *ALAT* alanine transaminase, *FAST* focused assessment with sonography for trauma, *CT* computed tomography, *n.s* no significance

Categorical data were reported as percentages of the corresponding subgroup; continuous data are presented as median (1.Q–3.Q)

^a^BIPS CT grade ≥ 4 subsumes CT findings of mesenteric contusion or haematoma, bowel wall thickening, adjacent interloop fluid collection, active bleeding, and pneumoperitoneum

^b^Sorted in the order of groups

**p-value of the ANOVA for continuous data and Pearson’s *χ*^2^ test for dichotomous data)

**Fig. 2:**
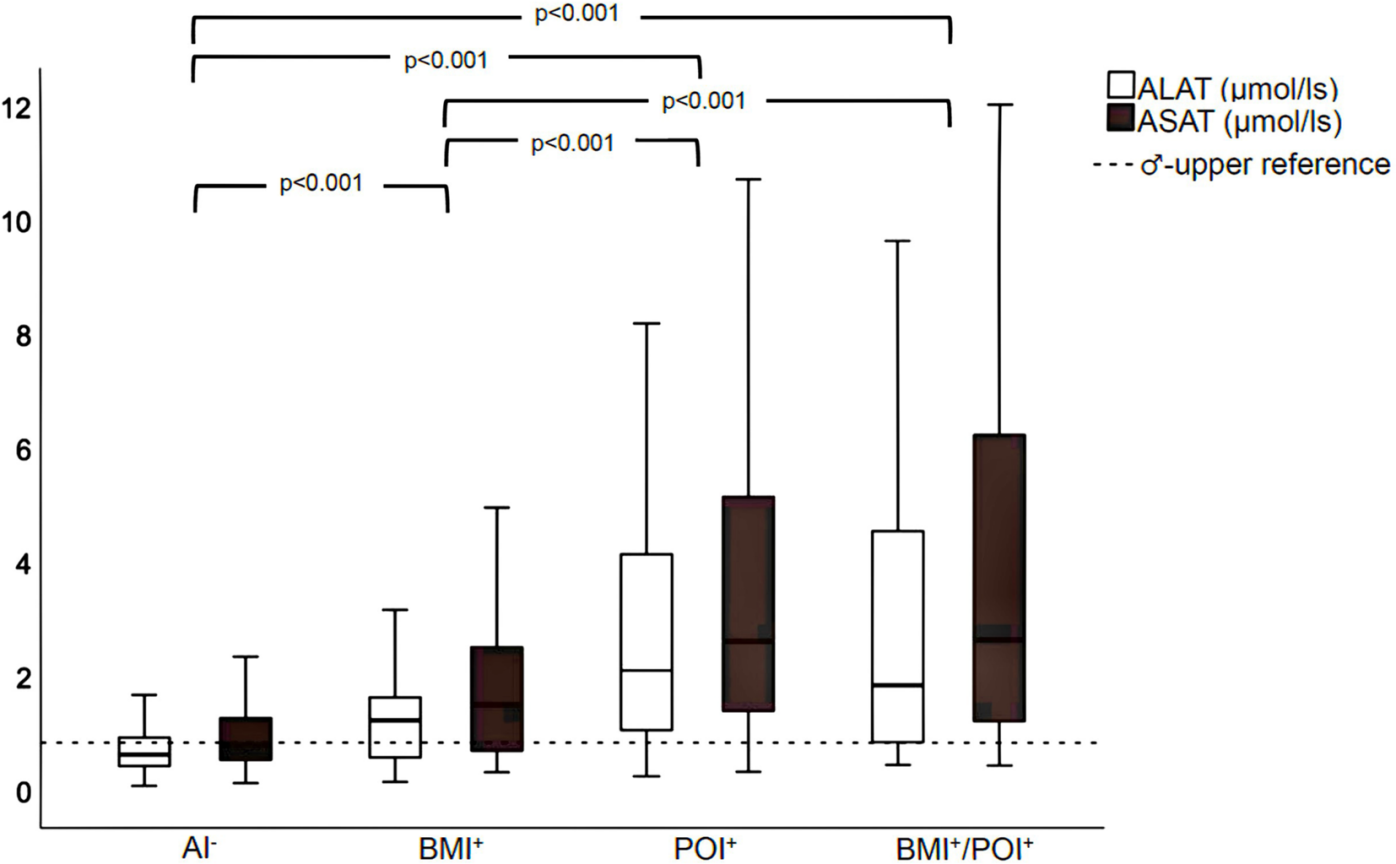
ANOVA subgroup comparison of transaminase values. *AI* abdominal injury, *BMI* bowel and mesenteric injury, *POI* parenchymatous organ injury, *ALAT* alanine transaminase, *ASAT* aspartate transaminase

Evidence of clinical signs was significantly more frequent in all three subgroups than in AI^−^. Signs within the three subgroups were significantly more frequent in the BMI^+^ and the BMI^+^/POI^+^ subgroups than in POI^+^. However, no significant differences were found between these groups. Concerning laboratory parameters, abdominal-specific values of ALAT and ASAT and the LAC level as indicators of mesenteric ischaemia were considered. ASAT and ALAT were significantly higher in the three AI^+^ subgroups than in the AI^−^ group. In detail, transaminases were significantly higher in BMI^+^/POI^+^ and POI^+^ compared to BMI^+^. However, no difference between BMI^+^/POI^+^ and POI^+^ could be seen (Fig. 2).

Increases in LAC were more frequent in all three subgroups. No significant distinctions could be calculated among the subgroups. Regarding imaging diagnostics, FAST and MSCT showed significantly more pathological findings across all three AI^+^ subgroups than AI^−^. However, there were no differences among the subgroups. Interestingly, the MSCT evidence of free fluid was evident more frequently in BMI^+^ and BMI^+^/POI^+^ compared to POI^+^ and the control group AI^−^. No differences were found between BMI^+^ and BMI^+^/POI^+^. The analysis of BIPS grade ≥ 4 showed the same results. In contrast, parenchymal damages, as detected by MSCT, were more frequent in POI^+^ and BMI^+^/POI^+^ compared to BMI^+^ and the control group AI^−^. No differences could be calculated between POI^+^ and BMI^+^/POI^+^.

Regarding associated injuries of other body regions, chest injury significantly goes hand in hand with injury of the upper parenchymal organs (BMI^+^/POI^+^: 87.5% and POI^+^: 87.9% in comparison to BMI^+^: 65.3% and AI^−^: 66.6%). Spine injuries and long-bone fractures did not show differences in prevalence among all groups. Pelvic fractures were significantly more frequent in all three subgroups than in the AI^−^ group. However, prevalence did not differ among the subgroups.

### Predictive power analysis of parameters: logistic regression

**Fig. 3:**
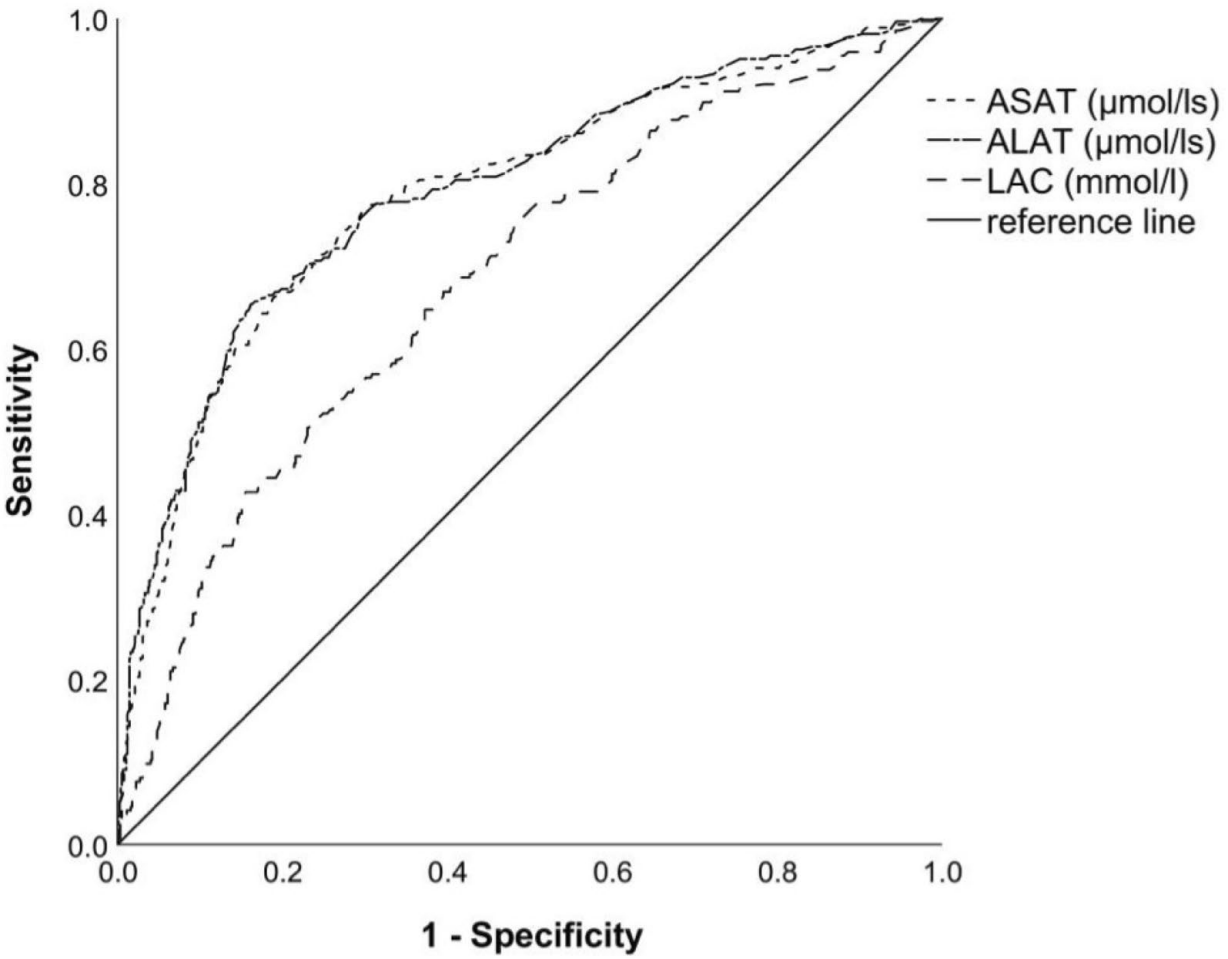
Exemplary ROC graph of areas under the curves (AUC) for the AI^+^ group. *ASAT* aspartate transaminase, *ALAT* alanine transaminase, *LAC* lactate

In the first step, ROC analysis calculated cut-off levels in the AI^+^ group and the BMI^+^ and POI^+^ sub-entities for specific abdominal laboratory values (ALAT, ASAT and LAC) (Fig. 3). The optimal cut-off levels calculated with Youden Indices are reported in Table 3.

**Table 3:** Optimal cut-off levels are calculated with Youden indices

Lab value	Upper reference	Youden indices		
		BMI	POI^+^	AI^+^
ALAT (µmol/ls)	♂ 0.83 ♀ 0.58	1.21	1.34	1.23
ASAT (µmol/ls)	♂ 0.83 ♀ 0.58	1.01	1.76	1.49
LAC (mmol/l)	♂ 2.4 ♀ 2.4	1.94	3.0	2.33

*ALAT* alanine transaminase, *ASAT* aspartate transaminase, *LAC* lactate, *BMI* bowel and mesenteric injury, *POI* parenchymatous organ injury, *AI* abdominal injury

Then, stepwise backward binary logistic regression modelling was performed to determine the values of predictive parameters for AI^+^ and the BMI^+^ and POI^+^ sub-entities. For this, only significant clinical examination parameters, pathological MSCT findings, cut-off values of ALAT, ASAT, and LAC, and the appearance of skeletal injuries in the main group and subgroup analyses were considered (Tables 1 and 2). Results of the FAST examination were excluded due to the high rate of missing data.

### Abdominal injury (AI^+^)

The results of stepwise backwards logistic regression (Table 4) identified four significant factors for the presence of a relevant abdominal injury (AI^+^). First, a pathological physical examination correlated more than threefold with the risk for an intraabdominal injury (OR 3.93). Second, evidence of pathological MSCT findings was expectably the most powerful parameter (OR 668.9) for AI^+^. Third, ALAT values ≥ 1.23 µmol/ls doubled the risk of the presence of an intraabdominal injury (sensitivity: 65.8%, specificity: 83.9%, PPV: 59%, NPV: 87%; OR 2.35). Fourth, long-bone fractures also represented a significant risk factor (OR 3.82) for abdominal participation.

**Table 4:** Results of multivariate analysis

	*n* = 843	Odds ratio	95% CI Odds ratio	*p*
Predictive parameters for AI^+^	Pathological abd. signs	3.9	1.6–10.0	0.004
	LAC ≥ 2.33 mmol/l			n.s
	ASAT ≥ 1.49 µmol/ls			n.s
	ALAT ≥ 1.23 µmol/ls	2.4	1.0–5.4	0.044
	CT: overall evidence of pathological signs (y/n)	668.9	258.9–1728.6	< 0.001
	Long-bone fracture	3.8	1.5–9.8	0.005
	Thorax injuries			n.s
	Pelvis injuries			n.s
Predictive parameters for BMI^+^	Pathological abd. signs	5.1		< 0.001
	LAC ≥ 2.33 mmol/l	2.1		0.044
	ASAT ≥ 1.49 µmol/ls			n.s
	ALAT ≥ 1.23 µmol/ls			n.s
	CT: overall evidence of pathological signs (y/n)	38.1	13.0–111.7	< 0.001
	Thorax injuries			n.s
	Pelvis injuries			n.s
Predictive parameters for POI^+^	Pathological abd. signs			n.s
	LAC ≥ 2.33 mmol/l			n.s
	ASAT ≥ 1.49 µmol/ls	4.3	2.3–8.0	< 0.001
	ALAT ≥ 1.23 µmol/ls			n.s
	CT: overall evidence of pathological signs (y/n)	222.9	101.3–490.4	< 0.001
	Thorax injuries	2.6	1.2–5.5	0.017
	Pelvis injuries			n.s

*LAC* lactate, *ASAT* aspartate transaminase, *ALAT* alanine transaminase, *CT* computed tomography, *AI* abdominal injury, *BMI* bowel and mesenteric injury, *POI* parenchymatous organ injury, *n.s.* no significance

### Bowel/mesenteric injury (BMI^+^)

Concerning risk factors for intestinal injuries (BMI^+^), examination of abdominal findings was significantly relevant, showing a more than fivefold increased risk (sensitivity: 71.1%, specificity: 87%, PPV: 36%, NPV: 96%; OR 5.12). Again, the best parameter for detecting BMI^+^ was MSCT (OR 38.06). A LAC level of ≥ 1.94 mmol/l significantly predicted a bowel and/or mesenteric injury (sensitivity: 84.4%, specificity: 47.2%, PPV: 14%, NPV: 97%; OR 2.23).

### Parenchymatous organ injury (POI^+^)

**Fig. 4:**
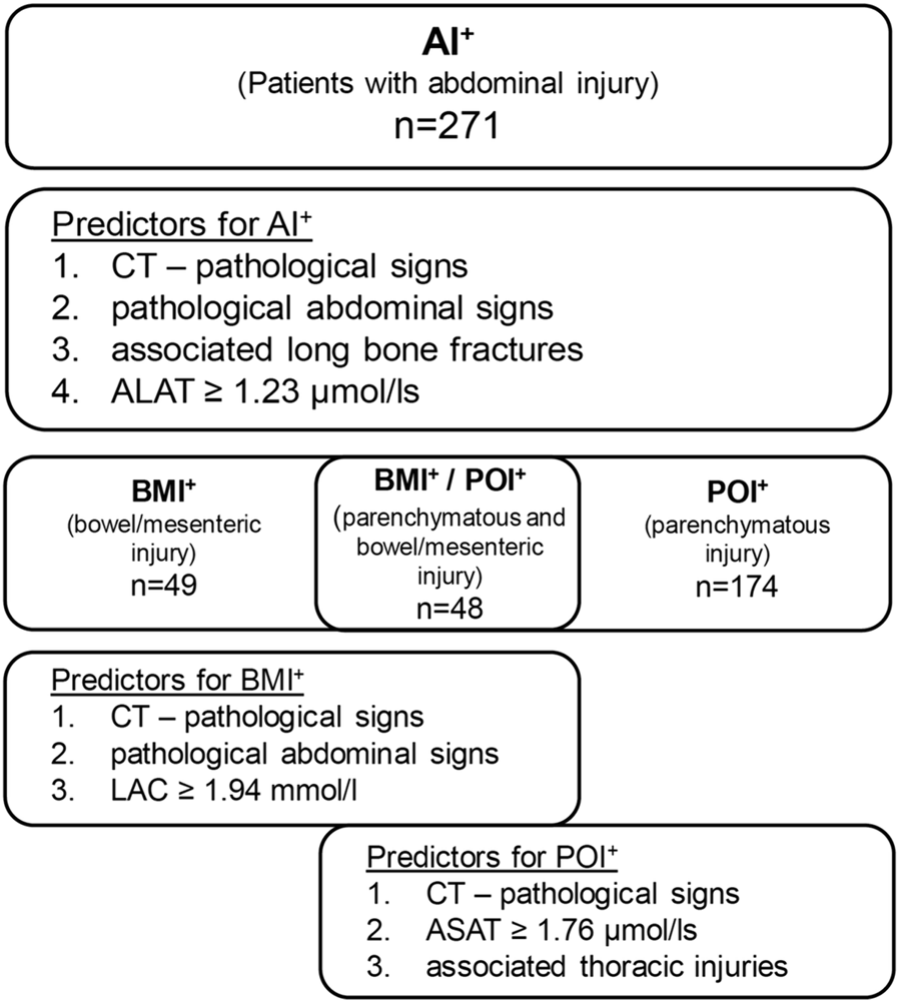
Predictive parameters for abdominal injury (AI^+^), bowel/mesenteric injury (BMI^+^), and parenchymatous injury (POI^+^) are sorted by OR height. *CT* computed tomography, *LAC* lactate, *ASAT* aspartate transaminase

The last stepwise backward regression could prove three significant predictive parameters for POI^+^. Again, the most powerful parameter was the MSCT with pathological findings (OR 222.9). However, ASAT levels ≥ 1.76 µmol/ls (OR 4.25) and concomitant thoracic injuries (OR 2.56) were significant risk factors for POI^+^, too. An ASAT value of ≥ 1.76 µmol/l had a sensitivity of 66.7% and specificity of 84.7%, with a PPV of 54% and an NPV of 90% for POI^+^. Figure 4 summarises all predictive parameters.

## Discussion

Despite the advances in technology for diagnosis and treatment, the identification of abdominal damages after blunt trauma in multiple-injury or polytraumatised patients still poses great challenges [29]. Accident victims are assessed by physical examination, laboratory tests, FAST and MSCT scan and explorative surgery to verify an abdominal involvement [6, 7]. In ER treatment, it is important to use simple and comprehensible parameters. Gad et al. [30] demonstrated in a cohort of 243 trauma patients with abdominal involvement that a delay in diagnosis and treatment increases mortality significantly. A contemporary and reliable diagnosis has a major role to play [30]. The present analysis provides predictive parameters for detecting relevant abdominal, bowel, mesenteric, or parenchymal upper abdominal organ injuries. Furthermore, the respective significance of the parameters was compared in a regression analysis.

Clinical examination remains an important diagnostic tool for detecting abdominal injuries. Our data show that an inconspicuous clinical examination is a safe predictor to filter out patients without abdominal injury. The logistic regression shows the clinical examination as a relevant parameter, especially for BMI^+^. Rostas et al. [31] confirmed its efficacy among patients with multiple injuries. A cohort of 803 patients could show a high sensitivity of 89.6% with an NPV of 97% to detect abdominal injuries [31]. However, our data could not reproduce such a high sensitivity (48.7%). Livingston et al. [32] focused on bowel injuries and found no significant correlation. Fakhry et al. [27] described a significant coherence between abdominal tenderness and perforated small-bowel injuries. Other authors described different scoring systems, using pathological abdominal signs as a significant risk factor to detect hollow-organ injuries [26, 28, 33].

Force impact to the abdomen may result from blunt, direct trauma or sudden deceleration [34]. In both situations, the increase of intraabdominal pressure may cause organic lesions [35]. Hence, specific laboratory values can be considered to indicate tissue damage [36]. Our study results show that increased transaminases as mitochondrial and cytoplasmic liver enzymes are reliable markers for abdominal injuries, especially for relevant liver or spleen parenchymal damage. In the literature, studies with smaller sample sizes have already indicated that abdominal trauma may cause an increase in transaminases [37, 38]. As early as 1991, Sahdev et al. [37] described a significant correlation between ALAT and ASAT serum levels > 130U/l (2.2 µmol/ls) and abdominal injury in 309 blunt trauma patients. Karaduman et al. [38] confirmed these results in a paediatric cohort of 87 individuals. As a final product of anaerobic glycolysis, LAC indicates mesenteric ischaemia [39]. Therefore, it can predict a hollow-viscus injury [39]. Previous studies have shown a direct correlation between LAC level and mortality in trauma patients [40, 41]. Raharimanantsoa et al. [28] described LAC as a significant predictor of intestinal injury. Our data emphasise their findings. LAC level ≥ 1.94 mmol/l increased the risk of BMI^+^ by more than twice. In contrast, Bekker et al. [14] could not confirm a correlation between the extent of a hollow-organ injury and the lactate level. Nowadays, the MSCT represents the gold standard of routine ER procedures in modern trauma centres for diagnosing polytraumatised patients [42]. Due to its high sensitivity and specificity, the high value of MSCT in diagnosing blunt-force abdominal injuries has been confirmed in many studies [18–20, 28, 34]. Even our data emphasise MSCT as the most powerful diagnostic tool, with a sensitivity of 94.8% and a specificity of 98%. Inconclusive findings such as free fluid may indicate a bowel and/or mesenteric injury. Active bleeding and pneumoperitoneum are definite findings in cases of a hollow-viscus injury [29]. Different authors developed scoring systems based on CT to filter out occult bowel lesions [26, 43]. Faget et al. [43] described a sensitivity of 96.4%, a specificity of 91.5% and an NPV of 99.6% in their scoring system. Keller et al. [44] verified the high value of the Faget score in their retrospective study from 2021. McNutt et al. [26] included WBC count, the result of clinical findings, and CT in their BIPS. In 2018, Zingg et al. [45] reviewed the BIPS for its validity and described that the use of the BIPS would lead to many diagnostic examinations without the BMI being detected. However, Fakhry et al. [27] identified a false-negative MSCT rate of 13% to detect perforated small-bowel injuries in a large multi-institutional study involving 275.557 trauma patients. However, MSCT should not be the only diagnostic tool for diagnosing BMI^+^ [27]. Our univariate analysis confirmed additional significant correlations of different CT signs, namely BIPS CT criteria ≥ 4pts and evidence of free fluid and parenchymal damage with AI^+^, BMI^+^, and POI^+^. Overall, the evidence of any pathological MSCT finding appears in all three regressions as the highest valuable risk factor.

Associated injuries could give information about potential abdominal involvement [46]. Thoracic and pelvic injuries, as well as long bone fractures, are significantly more common in those with AI^+^. However, only thoracic injuries for the upper parenchymatous organs (POI^+^) and long-bone fractures for abdominal injuries (AI^+^) can be proven as risk factors in the logistic regression. Raharimanantsoa et al. [28] included long-bone fractures in their scoring model to predict a relevant bowel or mesenteric injury. We could not reproduce such a relationship. In 2002, Demetriades et al. [47] described a correlation between severe pelvic injuries and liver injuries in a cohort of 16,000 trauma patients. Swaid et al. [48] could not reconstruct this relationship in a paediatric population. Our study showed an unspecific relationship between pelvic injuries and AI^+^. Thoracic injuries are frequently described as a risk factor for abdominal injuries, especially of the parenchymatous upper-abdominal organs [49, 50]. We could confirm these results in our univariate as well as multivariate analyses.

In summary, reliable predictors could be demonstrated for AI^+^, BMI^+^ and POI^+^ injury entities. In our view, the most difficult ambiguous subgroup was the BMI^+^/POI^+^ group. A slight spleen or liver injury could mask a hollow viscus injury with free fluid in the abdomen. Strict attention to the occurrence of further predictors could help. If uncertainty about diagnosis persists after all examinations, clinical monitoring and repeated FAST or MSCT in a tight timeframe is recommended. A recently published retrospective study by Lannes et al. [11] showed the increased predictive power of repeated short-term CT for detecting occult blunt bowel and mesenteric injuries. Furthermore, diagnostic laparoscopy may be useful in ruling out BMI^+^ promptly [51, 52].

Our study has several limitations, mostly due to the retrospective nature of the study and the collection from a single centre. The accuracy was affected by errors in documentation in the patient records and trauma registry. During the performance of an emergency MSCT, considerable artefact formation (attached arms) could have occurred, which may have been misinterpreted and led to a higher number of abdominal injuries. Sedation or unconsciousness at admission to the ER may have compromised the physical examination. But in cases of polytrauma, depression in a state of consciousness and MSCT with attached arms are not rare. Accordingly, these patients were included. Finally, given the relatively long study period of 13 years, it must also be stated that technical innovations such as more powerful imaging in higher-resolution sonography and computer tomography have certainly contributed to diagnostic certainty over time, resulting in a potential bias. In future analysis, the presented reliable predictors (Fig. 4) for the injury entities require further assessment and prospective validation.

## Conclusions

This study found reliable risk factors for abdominal, parenchymal, bowel and mesenteric injury. The presented risk factors could be well explained by the anatomy of the trunk and existing studies. The position of the parenchymatous organs in the lower thorax could explain the significant relationship between damage to the liver and spleen (POI^+^) and thoracic injury. It can be assumed that a relevant abdominal impact leads to a release of hepatic enzymes as a result of micro-trauma, also in cases of macroscopically undetectable liver injuries. Therefore, transaminases could predict POI^+^ and all abdominal injuries (AI^+^). As a product of anaerobic glycolysis and a marker of a mismatch with oxygen demand, LAC predicts BMI^+^. The pathological MSCT is the most reliable predictive parameter for all entities. However, it is necessary to include further relevant parameters, such as pathological physical examination, in the diagnosis of abdominal trauma.

## Data Availability

The datasets generated during and/or analysed during the current study are available throughout the manuscript.
